# Possible significance of hemodynamic and immunomodulatory effects of early stress-dose steroids in cardiac arrest

**DOI:** 10.1186/s13054-016-1384-4

**Published:** 2016-07-20

**Authors:** Spyros D. Mentzelopoulos, Nicolas Mongardon, Theodoros Xanthos, Spyros G. Zakynthinos

**Affiliations:** Department of Intensive Care Medicine, University of Athens Medical School, Evaggelismos General Hospital, 45-47 Ipsilandou Street, GR-10676 Athens, Greece; Service d’anesthésie et des réanimations chirurgicales, Réanimation chirurgicale cardio-vasculaire, DHU A-TVB, Hôpitaux Universitaires Henri Mondor, Assistance Publique des Hôpitaux de Paris, Créteil, France; Université Paris Est Créteil, Faculté de Médecine, Créteil, France; INSERM U955, équipe 3 «Stratégies pharmacologiques et thérapeutiques expérimentales des insuffisances cardiaques et coronaires», Créteil, France; School of Medicine, European University Cyprus, Nicosia, Cyprus

In an interesting randomized clinical trial (RCT), Donnino et al. [1] studied a mixed out-of-hospital cardiac arrest and in-hospital cardiac arrest (IHCA) population and found no hydrocortisone versus placebo hemodynamic or in-hospital outcome benefit. In the hydrocortisone group, the median time to study intervention was 9.9 h after return of spontaneous circulation (ROSC) [1]. This time lag probably exceeds the therapeutic window for the prevention of detrimental episodes of early post-resuscitation hypotension [2] through a mean arterial pressure (MAP)-stabilizing effect of steroids [3, 4].

Analyses of pooled post-resuscitation shock data from our IHCA vasopressin-steroids-epinephrine (VSE) RCTs [3, 4] also showed no between-group differences in the time to, or proportions of, discontinuation of vasopressors, and post-ROSC day 1 hemodynamic support (Table 1). However, VSE patients had higher, early post-ROSC systolic arterial pressure (SAP) and MAP during post-resuscitation follow-up [3, 4]. This reflected an improved hemodynamic response to similar vasopressor support titrated to a “wide” MAP range of 70–100 mmHg [4].

**Table 1:** Pooled results (from [3] and [4]) on early post-enrollment hemodynamics in survivors for ≥4 h with post-resuscitation shock

Survivors for ≥4 h with post-resuscitation shock^a^	VSE group (*n* = 103)	Control group (*n* = 88)	*P* value
Time to discontinuation of vasopressors (days), median (IQR)^b^	4 (2–8)	3 (2–6)	0.86
Discontinuation of vasopressors during follow-up, *n* (%)	43 (41.7)	34 (38.6)	0.77
Estimated cumulative vasopressor dose (μg/kg) over the first 24 h post-ROSC, median (IQR)^c,d,e^	552 (216–1225)	629 (321–1236) (*n* = 87)	0.15
Cumulative 24-h post-ROSC fluid balance (mL), mean ± SD	2168 ± 2398 (*n* = 78)	2034 ± 2198 (*n* = 60)	0.74
SAP >90 mmHg within 15–20 min post-ROSC, *n* (%)	76 (80.9) (*n* = 94)	40 (55.6) (*n* = 72)	0.001
At least 1 recorded/analyzed MAP value >80 mmHg over day 1, *n* (%)	82 (80.4) (*n* = 102)	35 (42.2) (*n* = 83)	<0.001
ALS duration (min), median (IQR)	10 (6–16)	12 (6–20)	0.11
	SAP >90 mmHg (*n* = 116)	SAP ≤90 mmHg (*n* = 50)	*P* value
Survival to hospital discharge with CPC score of 1 or 2, *n* (%)	23 (19.8)	3 (6.0)	0.02
	MAP >80 mmHg (*n* = 117)	MAP ≤80 mmHg (*n* = 68)	*P* value
Survival to hospital discharge with CPC score of 1 or 2, *n* (%)	25 (21.4)	4 (5.9)	0.006

Data reported as *n* (%) were analyzed with the Fisher’s exact test; data reported as median (IQR) were analyzed with the Mann-Whitney exact *U* test; and data reported as mean ± SD were analyzed with the independent samples *t* test

^a^Defined as sustained (>4 h), new post-arrest circulatory failure or post-arrest need for ≥50 % increase in any pre-arrest vasopressor/inotropic support targeted to MAP >70 mmHg [3, 4]

^b^Defined as number of days from study enrollment until the first circulatory failure-free day; the latter corresponds to a sequential organ failure assessment (SOFA) circulatory subscore <3; in both studies [3, 4], the SOFA score was determined daily through follow-up days 1–60 post-randomization

^c^With respect to [3]: average daily infusion rates (IRs) of vasopressors were already available as they were calculated by the investigators who conducted the follow-up; corresponding results were reported in the supplement of the originally published article. Consequently, Day 1 dose of a vasopressor (VD) (μg/kg) = average daily IR (μg/kg/min) × 1440 (min)

^d^With respect to [4]: for patients with IR data (μg/kg/min) available at 20 min post-ROSC (IR20M), 4 h post-ROSC (IR4H), and 24 h post-ROSC (IR24H) (*n* = 108), VD (μg/kg) was estimated as follows: VD = average (IR20M; IR4H) × 240 (min) + average (IR4H; IR24H) × 1200 (min). For patients with available IR20M and IR4H (*n* = 39), VD (μg/kg) was estimated as follows: VD = average (IR20M; IR4H) × 240 (min) + IR4H × (number of min until death after the completion of 4-h survival). For patients with available IR20M only (*n* = 1), VD (μg/kg) was estimated as follows: VD = IR20M × (number of min until death)

^e^For both [3] and [4], total day 1 VD (μg/kg) was calculated as follows [1]: VD = norepinephrine (μg/kg) + dopamine/2 (μg/kg) + epinephrine (μg/kg)

*ALS* advanced life support, *CPC* cerebral performance category, *IQR* interquartile range, *MAP* mean arterial pressure, *ROSC* return of spontaneous circulation, *SAP* systolic arterial pressure, *SD* standard deviation, *VSE* vasopressin-steroids-epinephrine

Recordings of “early post-ROSC SAP >90 mmHg” (i.e., “absence of early post-resuscitation hypotension” [2]) and “≥1 recorded/analyzed, day 1 MAP value of >80 mmHg [2]” were significantly more frequent in VSE patients than controls. Importantly, such SAP/MAP levels corresponded to more frequent survival to hospital discharge with favorable neurological outcome [4] (Table 1).

Early post-resuscitation hemodynamics of VSE patients could be partly attributable to the steroids-vasopressin combination during cardiopulmonary resuscitation (CPR) [3, 4]. However, a previously postulated major CPR-VSE effect, i.e., shorter advanced life support duration [4], possibly leading to attenuated post-resuscitation cardiovascular dysfunction was not clear in the current subgroup analysis (Table 1). Hence, according to the short (i.e., 24 min) half-life of vasopressin, we propose that the more frequent day 1 MAP >80 mmHg was largely due to a post-ROSC steroid-induced augmentation of vascular responsiveness to vasopressors [3, 4]. A mediation analysis of VSE outcome benefit through day 1 MAP is warranted. Analysis of day 1 MAP data from the study by Donnino et al. might causally link between-RCT differences in corticosteroid timing with differences in survival/neurological outcome results [1, 3, 4].

Post-resuscitation disease is a “sepsis-like” syndrome. In sepsis, acute kidney injury severity is associated with mortality and elevated interleukin (IL)-6. Furthermore, high post-ROSC IL-6 is associated with organ dysfunction and poor long-term outcomes [5]. Notably, post-resuscitation hydrocortisone has been associated with reduced IL-6 levels [1, 3], and VSE patients versus controls had more renal failure-free days [3, 4].

Conclusively, available evidence prompts toward further evaluation of early, stress-dose steroids in cardiac arrest.

## Abbreviations

CPR, cardiopulmonary resuscitation; IHCA, in-hospital cardiac arrest; IL, interleukin; MAP, mean arterial pressure; RCT, randomized clinical trial; ROSC, return of spontaneous circulation; SAP, systolic arterial pressure; VSE, vasopressin-steroids-epinephrine
